# Five-year follow-up of angiographic disease progression after medicine, angioplasty, or surgery

**DOI:** 10.1186/1749-8090-5-91

**Published:** 2010-10-26

**Authors:** Jorge Chiquie Borges, Neuza Lopes, Paulo R Soares, Aécio FT Góis, Noedir A Stolf, Sergio A Oliveira, Whady A Hueb, Jose AF Ramires

**Affiliations:** 1 Heart Institute (InCor) University of São Paulo Medical of School, São Paulo, Brazil

## Abstract

**Background:**

Progression of atherosclerosis in coronary artery disease is observed through consecutive angiograms. Prognosis of this progression in patients randomized to different treatments has not been established. This study compared progression of coronary artery disease in native coronary arteries in patients undergoing surgery, angioplasty, or medical treatment.

**Methods:**

Patients (611) with stable multivessel coronary artery disease and preserved ventricular function were randomly assigned to CABG, PCI, or medical treatment alone (MT). After 5-year follow-up, 392 patients (64%) underwent new angiography. Progression was considered a new stenosis of ≥ 50% in an arterial segment previously considered normal or an increased grade of previous stenosis > 20% in nontreated vessels.

**Results:**

Of the 392 patients, 136 underwent CABG, 146 PCI, and 110 MT. Baseline characteristics were similar among treatment groups, except for more smokers and statin users in the MT group, more hypertensives and lower LDL-cholesterol levels in the CABG group, and more angina in the PCI group at study entry. Analysis showed greater progression in at least one native vessel in PCI patients (84%) compared with CABG (57%) and MT (74%) patients (p < 0.001). LAD coronary territory had higher progression compared with LCX and RCA (P < 0.001). PCI treatment, hypertension, male sex, and previous MI were independent risk factors for progression. No statistical difference existed between coronary events and the development of progression.

**Conclusion:**

The angioplasty treatment conferred greater progression in native coronary arteries, especially in the left anterior descending territories and treated vessels. The progression was independently associated with hypertension, male sex, and previous myocardial infarction.

## Introduction

The frequency of progression of atherosclerosis in native coronary arteries in patients with established coronary artery disease (CAD) treated either with modern revascularization strategies or by current standard optimal medical therapy alone is unknown. Most progression occurs silently, without worsening symptoms or clinical events, and consequently, the prognostic significance of coronary progression, particularly in asymptomatic patients is uncertain [1,2]. The clear contrast between the occurrences of a clinical event with the slow progression of vascular lesions suggests the existence of different factors responsible for each condition [3,4].

Although the major concern of any revascularization treatment for CAD is its durability, few studies have given long-term angiographic follow-up results and are concerned with occlusion of the coronary bypass graft or restenosis of a treated lesion [5,6]. Accordingly, to date, few studies have investigated the predictors of chronologic native coronary atherosclerosis progression based on coronary angiography data in patients with treated stable multivessel CAD, including optimal medical therapy alone [7,8]. This post-hoc analysis of the MASS II trial comparatively describes the long-term angiographic native CAD progression in nonrevascularized or distal coronary lesions during the 5 years after medical treatment (MT), by-pass surgery (CABG), or percutaneous coronary intervention (PCI) and evaluated the predictors of native CAD progression in this setting. Also, we assessed whether the progression of native CAD was associated with subsequent clinical coronary events.

## Patients and Methods

### Study Design and Patient Population

The Medicine, Angioplasty, or Surgery Study (MASS-II) is a prospective, randomized, single-center study that compared medical, surgical, and angioplasty treatment in patients with symptomatic multivessel coronary artery disease and preserved left ventricular function. Details of the MASS II design, study protocol, patient selection, and inclusion criteria have been reported previously [9]. Briefly, patients with angiographically documented proximal multivessel coronary stenosis of > 70% by visual assessment and documented ischemia were considered for inclusion. Ischemia was documented by either stress testing or the typical stable angina assessment of the Canadian Cardiovascular Society (CCS) (Class II or III). Patients were enrolled and randomized if the surgeons, attending physicians, and interventional cardiologists agreed that revascularization could be attained by either strategy. Of 611 patients randomized between May 1995 and May 2000, 392 have undergone a new angiography after 5-year follow-up. The present report compared the atherosclerotic native coronary progression in those patients stratified according to the treatment received.

Patients gave written, informed consent and were randomly assigned to each treatment group. The Ethics Committee of the Heart Institute of the University of São Paulo Medical School in São Paulo, Brazil approved the trial, and all procedures were performed in accordance with the Helsinki Declaration.

Clinical criteria for exclusion included refractory angina or acute MI requiring emergency revascularization, ventricular aneurysm requiring surgical repair, left ventricular ejection fraction < 40%, a history of PCI or CABG, single-vessel disease, and normal or minimal CAD. Patients were also excluded if they had a history of congenital heart disease, valvular heart disease, or cardiomyopathy; if they were unable to understand or cooperate with the protocol requirements or to return for follow-up; or if they had left main coronary artery stenosis ≥ 50%, or suspected or known pregnancy or another coexisting condition that was a contraindication to CABG or PCI.

### Treatment Intervention

In the MASS II Trial, all patients were placed on an optimal medical regimen consisting of a stepped-care approach using nitrates, aspirin, beta-blockers, calcium channel blockers, angiotensin-converting enzyme inhibitors, or a combination of these drugs, unless contraindicated. Lipid-lowering agents, particularly statins, were also prescribed, along with a low-fat diet, on an individual basis with the objective of keeping low-density lipoprotein cholesterol < 100 mg/dL. Antihypertensive drugs were used according to the physicians' judgment. For diabetic treatment, sulfonylurea, insulin, and metformin were used with the main objective of keeping fasting glucose lower than 140 mg/dL. The medications were provided for free by the Heart Institute. Patients were then randomized to continue with aggressive medical therapy alone or to undergo PCI or CABG concurrently with MT.

Requirements were to perform optimal coronary revascularization in accordance with current best practices for both PCI and CABG. Equivalent anatomical revascularization was encouraged but not mandatory.

For patients assigned to PCI, the procedures were performed within 3 weeks after randomization. Devices used for catheter-based therapeutic strategies were left to the discretion of the operator and included stents, lasers, directional atherectomy, rotablator, and balloon angioplasty. Angioplasty was performed according to a standard protocol [8] that included administration of aspirin before the procedure. Glycoprotein IIb/IIIa agents were not used. Successful revascularization in the PCI group was defined as a residual stenosis of < 50% reduction in luminal diameter with thrombolysis in myocardial infarction (TIMI) flow grade 3.

For patients assigned to CABG, the procedures were performed within 12 weeks after randomization. Complete revascularization was accomplished if technically feasible, with saphenous vein grafts, internal mammary arteries, and other conduits, such as radial or gastroepiploic arteries. Standard surgical techniques [9] were used with patients under hypothermic arrest with blood cardioplegia. No off-pump CABG was performed.

### Angiographic Analysis

Coronary angiographies were performed with the Sones or Seldinger techniques in all 392 patients after enrollment and after 5 years of follow-up and were evaluated by visual assessment. Angiograms of the left and right coronary arteries were carried out in 6 to 8 projections, including half-axial projections. Two projections (in the majority of orthogonal projections) best representing the segments and stenoses to be analyzed were selected for further processing. All angiograms were recorded in a special protocol, allowing the repetition of the second angiogram in exactly the same projections, and by this, assuring optimal comparison between the 2 angiograms 5 years apart. Ten minutes before angiography, patients received 10 mg of isosorbide dinitrate sublingually to achieve maximal vasodilatation of coronary segments and eccentric stenosis. For assessment of ventricular function, patients underwent contrast left ventriculography at baseline in the right anterior oblique projection, and ejection fraction was calculated by using the Dodge formula [10].

Two experienced independent cardiologists blinded to the identity and clinical characteristics of patients, visually selected coronary artery segments and stenosis to be analyzed from high-quality cineframes. The inclusion of segments followed the recommendations of the American Heart Association; segments < 1.0 mm in diameter and all those located distally to occlusions, opacities only by collaterals, were excluded from further analysis. Stenosis reduced > 50% in diameter was considered significant, and a lesion reduced < 50% was considered mild. A segment with stenosis < 20% was interpreted visually and not included in the analysis. Angiographic morphology was scored independently, and if discrepancies arose, a third observer joined in the judgment, and the stenosis morphology was classified by consensus. Interobserver agreement in the quantitative analysis of all significant stenosis was 92%.

Progression of coronary atherosclerosis was defined as a new stenosis of at least 50% in an arterial segment previously considered normal or an increase in the grade of previous stenosis of > 20%. Furthermore, new stenosis in a native artery distal to grafts using the same defined criteria as above was considered as progression of coronary disease. Due to the nature of the physiopathology of occlusion, occlusion in a native coronary or in an artery that had received intervention (graft placement or stents implanted) was not considered. Both non-target lesions and non-target vessels were analyzed on this study. Regarding the different blood flow between bypassed and non-bypassed vessels, we decided to analyze on the bypassed vessel, only the segment post anastomosis.

### Follow-up

Adverse and other clinical events were tracked through randomization. Patients were assessed with follow-up visits every 6 months for 5 years at the Heart Institute. Patients underwent a symptom-limited treadmill exercise test, according to a modified Bruce protocol, at baseline and every year until the end of the study, unless contraindicated. We considered exercise test results positive when exertional angina developed or when we observed an ST-segment with an abnormal depression (horizontal or down-sloping of 1 mm for men and 2 mm for women) at 0.08 s after the J point. Routine examinations included electrocardiography and routine blood tests every 6 months.

Symptoms of angina were graded according to severity, from 1 to 4 as previously defined [10]. Angina was considered refractory only when patients had been treated with full anti-ischemic therapies to their level of tolerance. Myocardial infarction was defined as the presence of significant new Q waves in at least 2 electrocardiographic (ECG) leads or symptoms compatible with MI associated with creatine kinase, MB fraction concentrations that were more than 3 times the upper limit of the reference range.

The predefined primary end point for this current report was cardiac-related death, incidence of stroke or cerebrovascular accident (CVA), Q-wave MI, or refractory angina requiring revascularization. The performance of a revascularization procedure was considered an end point for patients in any group. In such a manner, therapeutic PCI or CABG performed during an episode of unstable angina at any time during follow-up was considered an end point and was applied equally across all 3 arms of therapy.

### Statistical Analysis

Statistical analysis was performed with SPSS 13.0 software (SSPS Institute Inc., Chicago, IL). The qualitative variables were reported as frequencies and percentages and were compared using the Fisher exact test or the chi-square test. The quantitative variables are descriptively presented in tables containing the average, standard deviation, median, minimum, and maximum values and were compared using the Student *t *test or Wilcoxon's test. All analyses were based on the intention to treat principle, and statistical tests were 2-tailed. Cox's proportional hazards method was used to develop a multivariate model of 5-year progression rates, including variables like sex, age, hypertension, hyperlipidemia, previous myocardial infarction, medication used, diabetes, collateral circulation, angina status, degree of coronary disease, treatment allocation, and clinical events. A p value of < 0.05 was considered statistically significant.

## Results

### Patient features by treatment groups

Of the 611 randomized patients, 392 have completed 5-year angiographic follow-up. None were lost to follow-up. The remaining 219 patients had not undergone angiographic study due to death, physicians' decision based on clinical conditions, or patient refusal. Of the 392 subjects studied, 136 were allocated to the surgery group, 146 to PCI, and 110 to MT. The baseline characteristics were similar among randomized treatment groups, except for more smokers and statin users in the MT group, more hypertension patients and lower LDL-cholesterol levels in the CABG group, and more angina CF II or III and less use of calcium channel antagonist in the PCI group at study entry (Table 1).

**Table 1:** Baseline characteristics of patients who underwent follow-up coronary angiography

Characteristics	MT (*n *= 110)	PCI (*n *= 146)	CABG (*n *= 136)	p
Demographic profile				
Age, y	59 ± 9	60 ± 9	61 ± 10	0.147
Female (%)	29	35	26	0.286

Medical history (%)				
Current Smoker	32	27	31	0.018
Hypertension	55	60	63	0.016
Diabetes mellitus	35	29	42	0.090
CCS class I or III angina	79	92	88	0.012

Laboratory values, mmol/L				
Total cholesterol	224 ± 39	227 ± 49	210 ± 43	0.007
LDL cholesterol	151 ± 34	151 ± 88	140 ±37	0.032
HDL cholesterol	37 ± 9	38 ± 10	36 ± 10	0.600
Triglycerides	200 ± 136	189 ± 94	181 ± 109	0.348

Medications				
Beta-blockers	79	79	86	0.209
Calcium-channel antagonists	62	42	66	0.001
Long-acting nitrates	90	84	82	0.0195
ACE inhibitors	35	33	28	0.467
HMG-CoA reductase inhibitors	26	16	13	0.024
Aspirin	97	98	96	0.719
Oral Hypoglycemic agents	14	8	12	0.333
Insulin	16	16	11	0.649

Positive treadmill test %	75	72	71	0.766

Entry angiographic features				
Mean ejection fraction	66 ± 25	67 ± 17	66 ± 19	0.328
Double-vessel disease, %	46	45	60	0.654
Triple-vessel disease, %	54	55	50	0.648
Proximal LAD, %	88	90	91	0.232

Vessel Territory ≥ 70%, %				
Left anterior descending	89	93	95	0.062
Left circumflex	71	70	78	
Right coronary artery	71	68	85	

Risk factor control at 5 years				
Aspirin use, %	95	94	95	0.926
Lipid-lowering drug, %	78	81	66	0.009
Current smoker, %	22	16	12	0.023

Total Events				
New intervention	24.2	32.2	3.5	0.001
Acute myocardial infarction	6	11	6	0.224
Stroke	2	3	2	0.884
Angina at 5 years	45.2	22.8	25.8	0.001

MT = medical treatment; PCI = percutaneous coronary intervention; CABG=coronary artery bypass graft; LAD = left anterior descending artery; ACE = angiotensin-converting enzyme, HMG-CoA = 3-hydroxy-3methylglutaryl-coenzyme-a, LDL and HDL = high- and low-density lipoprotein, respectively.

At follow-up, aspirin use continues to be frequent among the 3 treatment groups (94 to 95%); the prevalence of current smoking was modest and decreased markedly from study entry to 5 years similarly in all 3 groups, and the use of lipid-lowering drugs increased by approximately 4-fold, yet, the CABG group received less than the other groups (Table 1). Patients treated with PCI were most likely to be free of anginal symptoms after 5 years of follow-up compared with those treated with MT or CABG (77%, 55%, and 74%, respectively, p < 0.001). Conversely, we observed a significant reduction in rates of positive tests for CABG (26%; p < 0.001), no difference in PCI group (36%; p = 0.122) and a significant increase in positive tests in the MT group (51%; p < 0.001) at the end of follow-up. At the end of follow-up, the use of beta-blockers decreased significantly in the CABG group, and increased in the MT group (MT, 87%; PCI, 75%; CAGB, 71%; p = 0.011). Also, the use of calcium channel antagonists increased significantly only in the MT group (p < 0.001), and the use of nitrates decreased significantly in the PCI and CABG groups (p < 0.001).

### Initial revascularization and clinic coronary events

On admission, 42% randomly assigned patients had double-vessel disease and 58% had triple-vessel disease. There were approximately 3.6 ± 0.8 lesions with stenosis > 50% per patient and no total occlusions were found. All patients assigned to CABG underwent CABG, but 6 patients assigned to PCI underwent CABG as their initial treatment, and 17 patients assigned to MT underwent PCI (one) or CABG (16) as their initial treatment due to refractory angina. Each patient who underwent CABG had an average of 3.3 ± 0.8 vessels bypassed. All intended vessels were grafted in 72% of patients. At least one internal thoracic artery was used for grafting in 90% of patients, and 2 internal thoracic arteries and one radial artery was used in 30% of patients. Among the patients assigned to the PCI group, an average of 2.2 ± 0.5 lesions was dilated. Multivessel PCI was performed in 72% of patients. Immediate angiographic success was achieved in 92% of patients in whom PCI was attempted; 60% of them received 2 or 3 stents, and only 11% received 1 stent, reaching a total of 71% of patients who received at least one. Complete revascularization (as defined by successful intervention in all major vessels with at least 70% stenosis) was achieved in 41% of patients.

The overall major adverse events at the 5-year follow-up by 1 of the 3 therapeutic strategies are shown in Table 1. Of note, the PCI group needed significantly more new intervention procedures compared with MT or CABG groups; and the MT group had more angina at 5-year follow-up.

### Native CAD progression at five years

At the lesion level, 5-year angiography revealed a total of 2483 nontreated segment vessels. Of them, 48% have had a progression lesion as defined. When we compared the treatment groups, we observed that in the PCI group, 60% of the lesions had progression compared with 35% and 48% in CABG and MT groups, respectively (p = 0.002). Additionally, the LAD coronary territory had a higher progression compared with that in LCX and RCA (P < 0.001) (Table 2). Considering the patients' level, 84% of PCI patients have had at least one native vessel with progression compared with 57% and 74% of patients who underwent CABG or MT (p < 0.001) (Table 3).

**Table 2:** Coronary progression in patients stratified by treatment and territory

Progression		Total	MT (n = 110)	PCI (n = 146)	CABG (n = 136)	P Value
Progression	Total - vessels (%)	31	27	44	17	< 0.001
	Progression RCA (%)	29*	22	37	12	< 0.001
	Progression LCX (%)	25*	21	35	8	< 0.001
	Progression LAD (%)	37*	25	48	20	< 0.001

Occlusion	Total - vessels (%)	18	20	16	18	0.412
	Occlusion RCA (%)	22^‡^	21	17	13	0.342
	Occlusion LCX (%)	14^‡^	10	13	15	0.242
	Occlusion LAD (%)	18^‡^	17	8	15	0.376

RCA=Right Coronary Artery; LCX=Left Circumflex Artery, LAD=Left Anterior Descending Artery.

* p = 0.002; ^‡ ^p = 0.056

**Table 3:** Baseline characteristics of patients with progression of native coronary artery at 5-year follow-up

Characteristics	Progression (*n *= 286)	Nonprogression (109)	p
Demographic profile			
Age, y	60 ± 9	60 ± 10	0.147
Female (%)	28	35	0.191
Medical history (%)			
Current Smoker	28	32	0.268
Hypertension	59	56	0.635
Myocardial infarction(yes/no)	68/77	32/23	0.052
Diabetes	34	37	0.615
CCS class I or III angina	86	90	0.297

Laboratory values, mmol/L			
Total cholesterol	222 ± 46	221 ± 46	0.964
LDL cholesterol	149 ± 39	147 ± 39	0.658
HDL cholesterol	37 ±10	38 ±10	0.078
Triglycerides	188 ± 115	190 ± 114	0.395

Medications			
Beta-blockers	74	78	0.247
Calcium channel antagonists	62	42	0.020
Long-acting nitrates	86	83	0.414
ACE inhibitors	31	34	0.564
HMG-CoA reductase inhibitors	20	15	0.335
Aspirin	94	96	0.331

Entry angiographic features			
Double-vessel disease, %	49	39	0.072
Triple-vessel disease, %	51	61	
Collateral circulation	38	53	0.011

Treatment Received, %			
PCI	45	23	
CABG	23	47	< 0.001
MT	32	30	

Total Events (yes, no)	76/71	24/29	0.397
New CABG, %	7	11	0.168
New PCI, %	13	9	0.252
AMI	8	5	0.252
Angina 5 years, (yes, no)	42	30	0.024

Abbreviations as in table 1

Table 3 depicts the clinical and angiographic risk variables among progression patients. Coronary progression was significantly associated only with a history of hypertension (p = 0.041), and a tendency toward fewer previous myocardial infarctions compared with nonprogression patients (p = 0.052). Interestingly, the distribution of the number of vessel disease revealed a significant pattern of more double-vessel than triple-vessel disease among progression patients, and opposite distribution in the nonprogression patients (p = 0.048). Also, the presence of less collateral circulation was associated with more coronary progression in the progression patients (p = 0.011). Of note, the progression was likely higher among patients who received incomplete revascularization and less likely to occur in treated LAD and LCX territories. An unexpected finding in our study is that no statistical difference was found in terms of coronary events and the development of the progression of CAD. Yet, patients with coronary progression had significantly more angina at 5-year follow-up (p = 0.024).

Next, Table 4 shows that the multivariate analysis (adjusting for the factors described in the statistical section) revealed male sex (OR = 1.961; CI 1.131-3.399), hypertension (OR = 1.961; CI 1.131-3.399), previous myocardial infarction (OR = 1.845; CI 1.099-3.096), and PCI treatment were independent predictive risk factors of native CAD progression at 5 years. The PCI treatment conferred a 4.8-fold and 2.1-fold increased risk compared with CABG or MT, respectively. On the other hand, the presence of collateral circulation (OR = 0.485; CI 0.266-0.882) was an independent protective factor against native CAD progression in patients with stable multivessel disease.

**Table 4:** Multivariate Cox proportion regression model for native coronary progression in patients with multivessel CAD disease who underwent CABG, PCI, or MT.

	Hazard ratio	CI 95%	p values
PCI vs. CABG	4.779	2.526 - 9.043	< 0.001
PCI vs. MT	2.096	1.144 - 3.840	0.017
Male/female	1.961	1.131 - 3.399	0.016
Previous MI	1.845	1.099 - 3.096	0.020
Hypertension	1.318	1.002 - 1.733	0.048
Collateral circulation (Yes/No)	0.485	0.266 - 0.882	0.009

PCI = percutaneous coronary intervention; CABG = coronary artery bypass surgery. MI = myocardial infarction. Adjusted for age, sex, total and LDL-cholesterol, number of vessel disease, diabetes, statins and ACE inhibitors used, angina status, clinical events, treatment allocated, previous MI, and presence of collateral circulation. P-value according to the log-rank test.

Finally, we analyzed separately the progression of native coronary artery to total occlusion, because we can not rule out that this process could have resulted from the procedure treatment complications, or by acute episodes, not necessarily related to the slow progression of vascular lesions itself. However, no significant difference was noted among the 3 treatments. We observed more total occlusion in males (OR = 1.72, P = 0.0078, CI 1.154-2.574) and in those patients who experienced a new myocardial infarction during their follow-up (OR = 2.48, P = 0.0006, CI 1.477-4.196).

## Discussion

The frequency of progression of native coronary arteries after graft replacement or percutaneous intervention has been previously studied with short-term follow-up with the main focus on revascularization failure (e.g., restenosis or graft occlusion). However, the predictors of progression of native nontreated coronary artery disease in patients with stable CAD after revascularization has been reported less. Of note, no previous study has compared the natural history of atherosclerosis progression in coronary segments without intervention or distal arteries during 5 years after the initial PCI, CABG, or MT alone, and evaluated the predictors of native CAD progression in this setting. Therefore, the MASS II trial provides a unique opportunity to follow the natural history of coronary disease progression in treated patients with stable multivessel disease. This report demonstrates that native lesion progression determined by sequential coronary angiography separated by a 5-year interval in at least one segment vessel after treatment is common (48%), and that patients who underwent CABG treatment were less likely to develop progression in a native coronary artery. The PCI treatment conferred a 4.8-fold and 2.1-fold increased risk compared with CABG or MT, respectively. Additionally, the progression was independently associated with hypertension, male sex, and previous myocardial infarction. Conversely, the presence of collateral circulation was an independent protective factor against native CAD progression. Intriguingly, progression in these lesions did not account for any of the major events.

The treatment for stable CAD by either PCI or CABG is commonly used and clinically effective in relief of ischemic symptoms. But because CAD is a chronic pathobiologic process with acute exacerbation, effective relief of symptoms by revascularization or by current medical treatment cannot prevent the ongoing progression of atherosclerotic disease. The natural history of atherosclerosis progression following revascularization procedures limits the long-term benefits of these procedures and requires continuation of risk management. Indeed, there is strong evidence that, overall, revascularization is not superior to medical treatment alone to prevent death or myocardial infarction in stable patients.

Others [11,12] have already demonstrated that hypertension, a well-know atherogenic risk profile, is a risk factor for CAD progression, as are lipid profile and diabetes. We found only hypertension as an independent predictive factor, concomitantly with male sex. The fact that we found no correlation between lipid profile or statin treatment in our study might be explained by the homogenous characteristic profile of our population. Surprisingly, diabetes mellitus also was not related to disease progression in our study. It is well known that diabetes is associated with increased risk of cardiovascular events and death. However, it remains unclear whether these associations with clinical events result from an effect on the progression of atherosclerosis or are a consequence of changes that might facilitate the development of an acute thrombotic disease event. We also should point out that only survivors were evaluated after 5 years. Indeed, higher mortality was found in diabetic patients [12,13], mainly when they received medical treatment compared with revascularization intervention strategies in the MASS trial [14]. Taken together, we can not rule out, therefore, that diabetic patients with higher progression rates might be those who died.

As mentioned above, the original design of the MASS trial did not allow us to address the issue of atherosclerosis progression as a mortality predictor. Therefore, a longer follow-up study is expected. Anyway, Waters *et al*[15], contrary to the CASS study [16], demonstrated that progression was a predictor of death, along with hypertension and low ventricular ejection fraction.

Our main goal was to compare the available treatments for multivessel CAD, because there is no consensus about the best strategy to prevent atherosclerotic disease progression. Gensini *et al*[17] demonstrated a higher progression of atherosclerosis in the medical treatment group, while in the CASS study, progression occurred mainly in the surgery group [16]. There is another study, however, that did not show any difference in atherosclerosis progression between medical and surgery treatment [18].

To our knowledge, the present study is one of the few evaluated prospectively, in a 5-year follow-up, of patients with multivessel CAD assigned randomly to 3 different kinds of treatment. We found an overall higher progression rate in LAD coronary territories, mainly in patients who underwent PCI. Moreover, PCI compared with CABG-treated vessels more likely developed progression, as did complete revascularization. Published data regarding this issue are conflicting. The INTACT study [19] reported that RCA territory was more greatly affected, while the CASS study [16] showed a significant increase in LAD territory progression. Indeed, in the surgery group, those who received mammary grafts in the LAD were less likely to have progression than patients who received a saphenous vein graft. The reason for this better evolution in patients undergoing CABG might be explained by the use of mammary grafts. Patients who received saphenous vein grafts in the LAD had similar progression rates as those in the PCI group (data not shown). Different patient selection, clinical protocols, and angiogram follow-up time could explain some of these discrepancies.

## Comment

The present study showed that patients who underwent PCI treatment were more likely to develop progression in native coronary arteries, than those undergoing CABG or MT, especially in the left anterior descending territories and in treated vessels over 5-year follow-up. Moreover, the progression was independently associated with hypertension, male sex and previous myocardial infarction. Yet, the presence of collateral circulation conferred a protective effect against progression.

## Study Limitations

Coronary angiography is not the best way to assess atherosclerosis progression, primarily because its does not measure atherosclerosis but rather the reduction in luminal caliber at the lesion site relative to adjacent reference arterial segments considered free of disease. Therefore, we might underestimate the results in current progression studies. Moreover, there was neither a quantitative coronary measurement nor an IVUS approach to study progression of atherosclerosis in these patients. In fact, the difficulties and variability between observers and even in the same observer on visual evaluation of angiographic progression are well known. Nevertheless, as in our study, decisions in clinical practice are determined visually. Indeed, Detre *et al*[20] demonstrated that the cardiologist could predict progression > 30% in a coronary segment by visual assessment. Anyway, in the present study, we tried to minimize the errors by having 2 blinded observers. Although 392 patients underwent 5-year angiographic follow-up, 36% of the enrolled patients were not studied. Definitely there is a bias in only evaluating progression in the survivors; the progression might be higher in the deceased patients. Next, regardless of advances in PCI with the use of pharmacological stents and GP IIb/IIIa inhibitors, multivessel CAD patients had the best results when they underwent CABG. New tools like angiotomography might better define the relation between progressions of coronary artery disease in multiarterial patients undergoing the different treatment strategies.

## Abbreviations

CAD: coronary artery disease; LAD: left anterior descending; LCX:left circumflex artery; RCA: right coronary artery; PCI: percutaneous coronary intervention; CVA: cerebrovascular accident; CABG: coronary artery bypass graft surgery; MI: myocardial infarction; MASS: Medicine, Angioplasty or Surgery Study trial.

## Competing interests

No potential conflict of interest relevant to this article was reported.

JCB has received scholarship from CAPES - Coordenação de Aperfeiçoamento de Pessoal de Nível Superior, and FAPESP - Fundação de Amparo à Pesquisa do Estado de São Paulo.

## Authors' contributions

All authors read and approved the final manuscript.

The authors had full access to the data and take full responsibility for its integrity. All authors have read and agree to the manuscript as written.

## References

[B1] Singh RN Progression of coronary atherosclerosis. Clues to pathogenesis from serial coronary arteriography Br Heart J 1984 52 4 451 461 10.1136/hrt.52.4.451 6477785 PMC481659

[B2] Vecht RJ Nicolaides EP Duffett A Cumberland DC Accelerated progression of coronary artery disease Br Med J (Clin Res Ed) 1987 295 6594 357 359 10.1136/bmj.295.6594.357 3115447 PMC1247212

[B3] van der Wal AC Becker AE Koch KT Piek JJ Teeling P van der Loos CM David GK Clinically stable angina pectoris is not necessarily associated with histologically stable atherosclerotic plaques Heart 1996 76 4 312 316 10.1136/hrt.76.4.312 8983676 PMC484541

[B4] Petch MC The progression of coronary artery disease Br Med J (Clin Res Ed) 1981 283 6299 1073 1074 10.1136/bmj.283.6299.1073 6794762 PMC1507518

[B5] Brower RW Laird-Meeter K Serruys PW Meester GT Hugenholtz PG Long term follow-up after coronary artery bypass graft surgery. Progression and regression of disease in native coronary circulation and bypass grafts Br Heart J 1983 50 1 42 47 10.1136/hrt.50.1.42 6602618 PMC481369

[B6] Skowasch D Jabs A Andrié R Lüderitz B Bauriedel G Progression of native coronary plaques and in-stent restenosis are associated and predicted by increased pre-procedural C reactive protein Heart 2005 91 4 535 536 10.1136/hrt.2004.037317 15772225 PMC1768800

[B7] Mack WJ Xiang M Selzer RH Hodis HN Serial quantitative coronary angiography and coronary events Am Heart J 2000 139 6 993 999 10.1067/mhj.2000.105702 10827379

[B8] Bruschke AV Kramer JR Bal ET Haque IU Detrano RC Goormastic M The dynamics of progression of coronary atherosclerosis studied in 168 medically treated patients who underwent coronary arteriography three times Am Heart J 1989 117 2 296 305 10.1016/0002-8703(89)90772-2 2916405

[B9] Hueb W Soares PR Gersh BJ César LAM Luz PL Puig LB Martinez EM Oliveira SA Ramires JAF The Medicine, Angioplasty, or Surgery Study (MASS-II): a randomized controlled clinical trial of 3 therapeutic strategies for multi-vessel coronary artery disease: 1-year results J Am Coll Cardiol 2004 43 1743 1751 10.1016/j.jacc.2003.08.065 15145093

[B10] Campeau L Grading of angina pectoris (Letter to the editor) Circulation 1976 54 522 523 947585

[B11] Sipahi L Tuzcu EM Schoenhgen P Wolski KE Nicholls SJ Balog C Crowe TD Nissen SE Effects of normal, pre-hypertensive, and hypertensive blood pressure levels on progression of coronary atherosclerosis J Am Coll Cardiol 2006 48 833 838 10.1016/j.jacc.2006.05.045 16904557

[B12] Ganguly SS Al-Shafaee MA Bhargava K Duttagupta KK Prevalence of prehypertension and associated cardiovascular risk profiles among prediabetic Omani adults BMC Public Health 2008 8 108 109 10.1186/1471-2458-8-108 18394173 PMC2386813

[B13] Paterson JC Mills J Lockwood CH The role of hypertension in the progression of atherosclerosis Can Med Assoc J 1960 82 2 65 70 14430848 PMC1937647

[B14] Hueb W Gersh BJ Costa F Lopes N Soares PR Dutra P Jatene F Pereira AC Góis AFT Oliveira SA Ramires JAF Impact of diabetes on five-year outcomes of patients with multivessel coronary artery disease Ann Thorac Surg 2007 83 1 93 99 10.1016/j.athoracsur.2006.08.050 17184637

[B15] Waters D Craven TE Lesperance J Prognostic significance of progression of coronary atherosclerosis Circulation 1993 87 1067 1075 8484829 10.1161/01.cir.87.4.1067

[B16] Alderman EL Corley SD Fisher LD BR Chaitman DP Faxon ED Foster T Killip JA Sosa MG Bourassa Five-year angiographic follow-up of factors associated with progression of coronary artery disease in the coronary artery surgery study (CASS) J Am Coll Cardiol 1993 22 1141 1154 10.1016/0735-1097(93)90429-5 8409054

[B17] Gensini GG Kelly AE Incidence and progression of coronary artery disease Arch Intern Med 1972 129 8127 8147 10.1001/archinte.129.5.814 5025902

[B18] Glaser R Selzer F Faxon DP Laskey WK Cohen HA Slater J Detre KM Wilensky RL Clinical Progression of Incidental, Asymptomatic Lesions Discovered During Culprit Vessel Coronary Intervention Circulation 2005 111 143 149 10.1161/01.CIR.0000150335.01285.12 15623544

[B19] Lichtlen PR Nikutta P Jost S Deckers J Wiese B Raffembeul W Anatomical progression of coronary artery disease in humans as seen by prospective, repeated, quantitative coronary angiography. Relation to clinical events and risk factors. The INTACT Study Group Circulation 1992 86 828 838 1516195 10.1161/01.cir.86.3.828

[B20] Detre KM Wright E Murphy ML Takaro T Observer agreement in evaluating coronary angiograms Circulation 1975 52 6 979 986 1102142 10.1161/01.cir.52.6.979

